# Obituary

**Published:** 1885-09

**Authors:** 


### OBITUARY.

On Saturday, June 27th, died Dr. Henry N. Guernsey, of Philadelphia.

Dr. Guernsey has been a well-known and consistent homœopathist for upward of half a century. An enthusiastic believer in the doctrines of Hahnemann, he lectured for many years in the Homœopathic Medical College of Pennsylvania as Professor of Obstetrics. He relinquished the chair in 1869 when that College "merged" into the Hahnemann Medical College of Philadelphia.

The Class of '69 will remember with affection and gratitude his earnest efforts to instruct them in all the mechanism of obstetrics, and at the same time to infuse into their minds a clear understanding of the more enlightened treatment of Homœopathy and a firm reliance upon its principles in meeting the many trying emergencies that arise in such cases.

Twenty years ago he was a frequent contributor to the different homœopathic journals. He is best known, however, for his "key-note system" of selecting the similar remedy and for his great work on obstetrics, the best book on the subject that has ever been issued in the homœopathic school of medicine. In that book he has considerably elaborated his key-note system.

He was filled with a strong missionary spirit in advancing the cause of Homœopathy, and sought by association and the influence of his own example to convert to the pure practice many who are homœopathists only in name.

By most of his patients he will be remembered in deep reverence and affection. A Member of the Class of '69.

